# The prevalence, burden and risk factors associated with chronic obstructive pulmonary disease in Commonwealth of Independent States (Ukraine, Kazakhstan and Azerbaijan): results of the CORE study

**DOI:** 10.1186/s12890-018-0589-5

**Published:** 2018-01-30

**Authors:** Damilya Nugmanova, Yuriy Feshchenko, Liudmyla Iashyna, Olga Gyrina, Kateryna Malynovska, Eljan Mammadbayov, Irada Akhundova, Nadezhda Nurkina, Luqman Tariq, Janina Makarova, Averyan Vasylyev

**Affiliations:** 1 Semey State Medical University, Almaty, Kazakhstan; 2 National Institute of Phthisiology and Pulmonology F.G. Yanovsky of NAMS, Kiev, Ukraine; 3 National Medical University n.a. O.O. Bogomoletz, Kiev, Ukraine; 4 GlaxoSmithKline, Kiev, Ukraine; 5 Scientific Research Institute of Lung Diseases, Baku, Azerbaijan; 6 GlaxoSmithKline, Dubai, UAE; 7 GlaxoSmithKline, Moscow, Russia; 8 GSK Russia Business Park “Krylatsky Hills”, 17, Krylatskaya Street, Building 3 (“Air”), 121614 Moscow, Russia

**Keywords:** Chronic obstructive pulmonary disease (COPD), Prevalence, Risk factors, Ukraine, Kazakhstan, Azerbaijan

## Abstract

**Background:**

In the Commonwealth of Independent States (CIS) countries the epidemiology of chronic obstructive pulmonary disease (COPD) is poorly characterized. The objective of this analysis is to present the prevalence, burden and risk factors associated with COPD in three CIS countries as part of the CORE study (Chronic Obstructive Respiratory Diseases), the rationale and design of which have been described elsewhere.

**Methods:**

A total of 2842 adults (≥18 years) were recruited (964 in Ukraine, Kiev, 945 in Kazakhstan, Almaty and 933 in Azerbaijan, Baku) between 2013 and 2015 during household visits. Two-step cluster randomization was used for the sampling strategy. All respondents were interviewed about respiratory symptoms, smoking status and medical history, and underwent spirometry with bronchodilator. COPD was defined as (i) “previously diagnosed” when the respondent reported that he/she had previously been diagnosed with COPD by a doctor, (ii) “diagnosed by spirometry” using the GOLD criteria (2011) based on spirometry conducted during the study (FEV_1_/FVC <  0.70), and (iii) “firstly diagnosed by spirometry”, when the patient had received the COPD diagnosis for the first time based on the spirometry results obtained in this study.

**Results:**

The prevalence of “previously diagnosed” COPD was 10.4, 13.8 and 4.3 per 1000, and the prevalence of COPD “diagnosed by spirometry” was 31.9, 66.7 and 37.5 per 1000 in Ukraine, Kazakhstan, and Azerbaijan, respectively. Almost all respondents with COPD were diagnosed for the first time during this study. A statistically significant relationship was shown between smoking and COPD in Kazakhstan (odds ratio, OR: 3.75) and Azerbaijan (OR: 2.80); BMI in Ukraine (OR: 2.10); tuberculosis in Ukraine (OR: 32.3); and dusty work in Kazakhstan (OR: 2.30). Co-morbidities like cardiovascular diseases and a history of pneumonia occurred significantly (*p* <  0.05) more frequently in the COPD population compared to the non-COPD population across all participating countries. For hypertension, this was the case in Ukraine and Azerbaijan.

**Conclusion:**

In CIS countries (Ukraine, Kazakhstan and Azerbaijan), the prevalence of COPD “diagnosed by spirometry” was significantly higher than the prevalence of previously diagnosed COPD. Compared to many other countries, the prevalence of COPD seems to be relatively low in CIS countries. Factors such as limited funding from the government; lack of COPD knowledge and the attitude within the population, and of primary care physicians; as well as low access to high-quality spirometry may play a role in this under-diagnosis of COPD. The information provided in this paper will be helpful for healthcare policy makers in CIS countries to instruct COPD management and prevention strategies and to allocate healthcare resources accordingly.

## Background

Chronic obstructive pulmonary disease (COPD) is a chronic respiratory disease with high worldwide prevalence, which is increasing, particularly in developing countries [1, 2]; moreover, it is associated with a high social burden. It was estimated in 2010, that over 230 million people living in urban areas (prevalence of 13.6%), and more than 153.7 million people living in rural areas (prevalence of 9.7%), are affected by COPD [2]. By 2010 COPD had become the third leading cause of death worldwide [3], and the majority of COPD-related deaths occurred in low– and middle-income countries [2].

COPD is characterized by chronic diffuse irreversible airflow obstruction involving mainly small airways. This condition is a growing cause of morbidity, disability, and mortality in both developed and developing countries that can be related to environmental exposures, smoking and respiratory infectious diseases [4]. Although the importance of early diagnosis is undisputed, patients with COPD often consult a physician at the late stages of the disease [5]. According to the European Respiratory Society (ERS), only 25% of cases are diagnosed at the early stages [6].

Previously published studies evaluating the prevalence of COPD have provided a range of estimates across different countries. The prevalence of previously diagnosed COPD (when diagnosis was made in the past and reported by a respondent) ranged from 18 per 1000 (Sweden) to 222 per 1000 (Russia); and the prevalence of COPD diagnosed by spirometry ranged from 37 per 1000 (United Arab Emirates) to 240 per 1000 (The Netherlands). In the multinational BOLD study the overall prevalence of COPD diagnosed by spirometry using a standardized approach was 193 per 1000 [7]. COPD prevalence estimates from across the globe are summarized in Table 1.

**Table 1:** Reported prevalence of COPD across different regions

Source and study location	Survey period	Estimated prevalence per 1000 (95% CI)		
		Previously diagnosed^a^	Diagnosed by spirometry^b^	Firstly diagnosed by spirometry^c^
Menezes AM et al. Montevideo, Uruguay (PLATINO study) [20]	2002	ND	197 (172–222)	ND
Menezes AM et al. Mexico City, Mexico (PLATINO study) [20]	2002	ND	78 (59–97)	ND
Doney B et al. USA [19]	2004–2011	ND	42 (40–43)	ND
Buist AS et al.12 sites across Europe, Asia, USA, Canada, South Africa and Australia (BOLD study) [7]	2005–2007	ND	193 (ND)	ND
Bárbara C et al. Lisbon, Portugal [28]	2006–2007	ND	142 (111–181)	ND
Danielsson P et al. Uppsala, Sweden (BOLD study) [34]	2006–2007	ND	162 (CI: ND)	ND
Minas M et al. Greece [15]	2006–2007	57 (CI: ND)	184 (CI: ND)	127 (CI: ND)
Soriano JB et al. Spain [16]	2007	ND	45 (24–66)	ND
Vanfleteren LE et al. Maastricht, Netherlands [23]	2007–2009	88 (CI: ND)	240 (CI: ND)	ND
Carlsson AC et al. Stockholm, Sweden [21]	2007–2011	18 (CI: ND)	ND	ND
Yoo KH et al. South Korea [17]	2008	ND	134 (CI: ND)	ND
Sousa CA et al. São Paulo, Brazil [25]	2008–2009	42 (31–54)	ND	ND
Al Zaabi A et al. Abu Dhabi, United Arab Emirates [22]	2009–2010	ND	37 (20–53)	ND
Lâm HT et al. Northern Vietnam [18]	2009–2010	ND	71 (CI: ND)	ND
Chuchalin AG et al. Russia (GARD Study) [26]	2010–2011	222 (212–232)	218 (195–245)	ND
Van Gemert F et al. Uganda [27]	2012	ND	162 (CI: ND)	ND
Tageldin MA et al. Middle East and North Africa (BREATHE study) [35]	2012	36 (35–37)	ND	ND

*COPD* – chronic obstructive pulmonary disease; *95% CI* – 95% confidence interval; *ND* – not determined

^a^Defined as: when diagnosis was made in the past and reported by a respondent

^b^Defined as: diagnosed and confirmed by spirometry conducted during the course of the study

^c^Defined as: diagnosed for the first time based on spirometry

More than half of those with chronic respiratory diseases live in low and middle income countries, including some Commonwealth of Independent States (CIS) countries [8], where there are no population-based epidemiology studies conducted to evaluate the prevalence of these diseases. The CORE study (Chronic Obstructive REspiratory diseases in CIS countries) has been conducted to bridge this gap. The aim of the study was to evaluate the country-specific point prevalence of COPD, bronchial asthma and allergic rhinitis in selected CIS countries in order to obtain a clear “epidemiological picture” of the disease. The rationale and design of the CORE study (including the key steps of the recruitment phase, inclusion and exclusion criteria, study population, demographic characteristics, employment status, education and marital status of participants and the questionnaires used in the study) have been described previously [9]. In this manuscript, data obtained on the prevalence and burden of COPD will be presented. In addition, the potential relationship between the presence of COPD and its risk factors will be assessed.

## Methods

### Study area and population

The CORE study is a multi-national, cross-sectional population-based epidemiological study conducted across major cities in Ukraine, Kazakhstan and Azerbaijan (Kiev, Almaty and Baku, respectively) from the first half of 2013 until the end of 2015. The study enrolled subjects who were ≥18 years old, had lived in the selected city for ≥10 years and provided written informed consent to participate in the study.

Subjects for whom spirometry could not be performed, and subjects who were not able to answer the study questionnaires (American Thoracic Society (ATS) Respiratory Symptoms Questionnaire, COPD Assessment Test (CAT™), Alcohol Intake, Tobacco Smoking Questions) or had any contraindication for spirometry or hypersensitivity to bronchodilator (Salbutamol) were excluded from the study. Contraindications for spirometry were established based on the judgment of the investigator and defined according to Cooper BG, 2011 [10] including absolute (myocardial infarction, ascending aortic aneurysm, pulmonary embolism, angina) or relative (thoracic/abdominal surgery; brain, eye, ear, nose or throat surgery; pneumothorax, haemoptysis, acute diarrhea, severe hypertension, confused/demented patients; patient discomfort, or infection control issue) contraindications.

### Case definition and severity

Diagnosis of COPD was established based on the study questionnaire and/or spirometry conducted without bronchodilator (pre-dose) and with bronchodilator (post-dose: 15–20 min after administration of 200–400 mcg Salbutamol (GlaxoSmithKline). Consequently, FEV_1_ (forced expiratory volume in one second) and FVC (forced vital capacity) were estimated during the spirometry using a standardized approach defined by Miller MR et al., 2005 [11]. Spirometry quality and results were regularly reviewed by members of the Study Executive Committee.

COPD was identified through three definitions:

1. Previously diagnosed: When self-reported by the respondent while completing the study questionnaire. Respondents were asked to answer the following question to identify “previously diagnosed COPD”:

*Has a doctor ever told you that you have chronic obstructive pulmonary disease (COPD)?*

*If YES, please, indicate number of exacerbations during the last year.*

2. Diagnosed by spirometry: confirmed by spirometry results based on the GOLD (Global Initiative for Chronic Obstructive Lung Disease) guideline (2011) (FEV_1_/FVC <  0.70). Spirometry was conducted as part of the study (Table 2).
3. Firstly diagnosed by spirometry: when the respondent was diagnosed with COPD for the first time based on spirometry outcomes.

**Table 2:** COPD diagnosis and severity based on spirometry outcomes (GOLD 20115)

Diagnosis	Post-dose parameters
No disease	FEV_1_/FVC ≥ 0.70
COPD GOLD stage:	
Stage I: Mild	FEV_1_/FVC < 0.70 FEV_1_ ≥ 80% predicted
Stage II Moderate:	FEV_1_/FVC < 0.70 50% ≤ FEV_1_ < 80% predicted
Stage III: Severe	FEV_1_/FVC < 0.70 30% ≤ FEV_1_ < 50% predicted
Stage IV: Very Severe	FEV_1_/FVC < 0.70 FEV_1_ < 30% predicted or FEV_1_ < 50% predicted plus chronic respiratory failure

*FEV*_1_ forced expiratory volume in one second; *FVC* forced vital capacity; respiratory failure: arterial partial pressure of oxygen (PaO_2_) less than 8.0 kPa (60 mmHg) with or without arterial partial pressure of CO_2_ (PaCO_2_) greater than 6.7 kPa (50 mmHg) while breathing air at sea level

As shown in Table 2, severity of COPD was investigated based on the GOLD guidelines (2011) [5] while using four categories for severity based on spirometry results only.

### Data collected

The data were collected from participants during household visits. Two-step cluster randomization (first step, administrative district; second step, street) was used for the sampling strategy. Districts and streets for household visits were selected by the Study Executive Committee. The interviewers visited households sequentially, starting with the first apartment of the first house in the selected street, and continuing in ascending order. At every household the interviewers assessed the eligibility of all inhabitants. Participants who provided their consent and were eligible to participate in the study provided their socio-demographic information and medical history, underwent weight/height measurement and spirometry with bronchodilator and completed the study questionnaires, as described previously [9].

Socio-demographic data were collected to describe the characteristics of the overall study population, including gender, age and ethnicity distribution, body mass index (BMI), smoking status and alcohol intake. COPD prevalence data were collected using the case definition described above and the impact of age, gender and severity were assessed. Additionally, the type and frequency of co-morbidities and any potential association between COPD and its related risk factors (i.e. smoking, BMI, alcohol intake, tuberculosis, dusty work, open fire cooking) were investigated.

The COPD Assessment Test (CAT™) and the modified Medical Research Council (mMRC) Dyspnoea Scale were used to assess additional characteristics of COPD in this study. All respondents answered the CAT™ and were assessed by the mMRC Dyspnoea Scale at the interview. The CAT™ is a validated short questionnaire for measurement of the impact of COPD on a patient’s health status [12]. It comprises eight items, each with a scoring range of 0–5. The total CAT™ score is derived based on the sum of responses given to the eight items with a range of 0–40. The mMRC Dyspnoea Scale was used as a simple grading system to assess the level of dyspnoea/shortness of breath in five categories from 0 to 4: 0 - Responder is not affected by shortness of breath, except when engaging in strenuous exercise; 1 - Responder has shortness of breath when walking briskly on flat ground or slightly uphill; 2 - Responder walks more slowly on flat surfaces than other people his/her age because of shortness of breath, or he/she has to stop to catch his/her breath when walking at his/her own pace on flat ground; 3 - Responder has to stop to catch his/her breath after walking around 100 m or after walking for a few minutes on flat ground; 4 - Responder’s shortness of breath prevents him/her from leaving home or he/she has shortness of breath when dressing or undressing.

### Statistical analysis

The point prevalence of COPD, (overall and separate stages/categories), is defined as the number of COPD individuals divided by total number of subjects included in the study, and is expressed as a number per 1000 for each country. Prevalence was calculated in the subject population with valid data. 95% confidence intervals (CI) were calculated for each frequency using the Clopper-Pearson method [13]. Odds ratios (OR) and 95% CI were calculated to estimate the statistical significance (*p*˂0.05) of associations between risk factors and COPD. Statistical analysis was performed using IBM SPSS Statistics software (IBM Corp., USA) version 21.0 and R software version 3.1.2 (R Core Team, Austria).

## Results

### Study sample and demographics

A total of 2842 adult subjects were included in the CORE study (964 in Ukraine, 945 in Kazakhstan and 933 in Azerbaijan). The majority of study population were women across the three countries: 58.2% in Ukraine; 63.2% in Kazakhstan and 58.3% in Azerbaijan. The mean age was slightly above 40 years old in all participating countries. As expected, the majority of participants were Caucasian in Ukraine (99.7%) and Azerbaijan (100%), and almost two-thirds of participants in Kazakhstan were Asian (62.8%). The mean BMI was at the boundary of overweight in Ukraine, (25.0 (5.1) kg/m^2^), and in Kazakhstan, (25.7 (5.1) kg/m^2^), while it was slightly higher in Azerbaijan (26.4 (5.3) kg/m^2^). Approximately one-third of participants were either current or past smokers (33.7% in Ukraine, 40.2% in Kazakhstan, 26.0% in Azerbaijan). Heavy alcohol consumption was reported by 53.4% of respondents in Ukraine, 44.8% in Kazakhstan and 22.7% in Azerbaijan. See Table 3.

**Table 3:** Demographic characteristics of respondents

		Ukraine	Kazakhstan	Azerbaijan
Gender	Male	403 (41.8%)	348 (36.8%)	389 (41.7%)
	Female	561 (58.2%)	597 (63.2%)	544 (58.3%)
	Total	964	945	933
Ethnicity	Asian	3 (0.3%)	593 (62.8%)	0
	Black	0	1 (0.1%)	0
	Caucasian	961 (99.7%)	349 (36.9%)	933 (100.0%)
	Other	0	2 (0.2%)	0
	Total	964	945	933
Age, years	Mean (SD)	40.7 (15.1)	42.5 (15.3)	40.7 (14.8)
	18–39 years old	482 (50.1%)	454 (48.0%)	467 (50.1%)
	40–64 years old	408 (42.4%)	423 (44.8%)	414 (44.4%)
	≥ 65 years old	72 (7.5%)	68 (7.2%)	52 (5.6%)
BMI, kg/m^2^	Mean (SD)	25.0 (5.1)	25.7 (5.1)	26.4 (5.3)
Overweight/obesity (BMI ≥ 25 kg/m^2^)	Overall population	437 (45.4%)	449 (47.6%)	511 (54.9%)
	Males	210 (52.1%)	165 (47.6%)	212 (54.5%)
	Females	227 (40.5%)	284 (47.6%)	299 (55.3%)
Smoking	Never smoked	629 (65.2%)	564 (59.7%)	690 (74,.0%)
	Current/past smoker	325 (33.7%)	380 (40.2%)	243 (26.0%)
Alcohol intake	Standard drinks^a^, mean (SD)	2.63 (4.15)	2.99 (7.54)	1.40 (2.82)
	Not at all	77 (8.0%)	240 (26.3%)	536 (57.4%)
	Moderate^b^	371 (38.6%)	263 (28.9%)	185 (19.8%)
	Heavy^b^	514 (53.4%)	408 (44.8%)	212 (22.7%)

*BMI* – body mass index; *SD* – standard deviation

^a^One drink was defined as 12 fluid ounces of regular beer (5% alcohol), 5 fluid ounces of wine (12% alcohol), or 1.5 fluid ounces of 80 proof (40% alcohol) distilled spirits. One drink contains 0.6 fluid ounces of alcohol

^b^Moderate alcohol consumption was defined as the consumption of up to 1 drink per day for women and up to 2 drinks per day for men. Heavy (or high-risk) drinking was defined as the consumption of more than 3 drinks on any day or more than 7 per week for women and more than 4 drinks on any day or more than 14 per week for men

### Prevalence of COPD

**Fig. 1:**
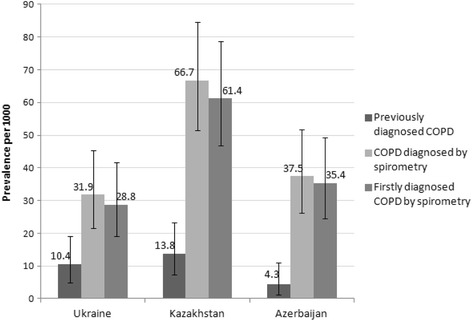
Point prevalence of COPD in the whole population. The prevalence was calculated per 1000 persons and expressed with 95% confidence intervals, for three COPD definitions: *previously diagnosed COPD* (when self-reported by the respondent while completing the study questionnaire); *COPD diagnosed by spirometry* (confirmed by spirometry results based on GOLD Guidelines (2011), i.e. FEV_1_/FVC <  0.70), and *firstly diagnosed COPD by spirometry* (when the respondent was diagnosed with COPD for the first time based on spirometry outcomes)

Subjects who fulfilled the definitions used in this study for COPD are shown in Table 4. Prevalence of previously diagnosed COPD was 10.4 (95% CI 5.0–19.1) per 1000 in Ukraine, 13.8 (95% CI 7.3–23.4) per 1000 in Kazakhstan, and 4.3 (95% CI 1.2–11.0) per 1000 in Azerbaijan. The estimated prevalence of COPD diagnosed by spirometry was higher among all participating countries compared to previously diagnosed COPD estimates: 31.9 (95% CI 21.7–45.3) per 1000 in Ukraine, 66.7 (95% CI 51.6–84.5) per 1000 in Kazakhstan, and 37.5 (95% CI 26.3–51.8) per 1000 in Azerbaijan. Almost all participating subjects were firstly diagnosed for COPD by spirometry. See Fig. 1.

**Table 4:** Number of respondents with COPD previously diagnosed and diagnosed by spirometry during the study

	Ukraine (*n* = 939)	Kazakhstan (*n* = 945)	Azerbaijan (*n* = 933)
Previously diagnosed COPD	*N* = 10 (1.0%)	*N* = 13 (1.4%)	*N* = 4 (0.4%)
Diagnosed by spirometry	*N* = 30 (3.2%)	*N* = 63 (6.7%)	*N* = 35 (3.8%)
Firstly diagnosed by spirometry	*N* = 27 (2.9%)	*N* = 58 (6.1%)	*N* = 33 (3.5%)
COPD severity by GOLD stage^a^:			
I (Mild)	13 (1.4%)	26 (2.8%)	13 (1.4%)
II (Moderate)	17 (1.8%)	37 (3.9%)	22 (2.4%)

GOLD Stage I (Mild): FEV_1_/FVC < 0.70 and FEV1 ≥ 80% predicted

GOLD Stage II (Moderate): FEV_1_/FVC < 0.70 and 50% ≤ FEV_1_ < 80% predicted

^a^There were no respondents diagnosed with COPD III or IV GOLD stages

**Fig. 2:**
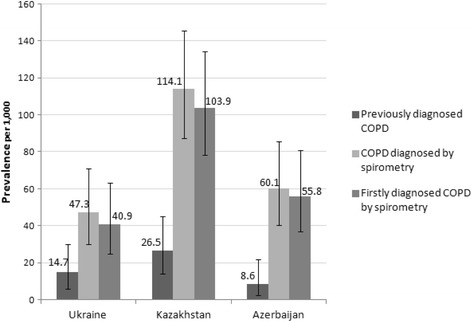
Point prevalence of COPD among respondents ≥40 years old. The prevalence was calculated per 1000 persons and expressed with 95% confidence intervals, for three COPD definitions: *previously diagnosed COPD* (when self-reported by the respondent while completing the study questionnaire); *COPD diagnosed by spirometry* (confirmed by spirometry results based on the GOLD guideline (2011), i.e. FEV_1_/FVC <  0.70), and *firstly diagnosed COPD by spirometry* (when the respondent was diagnosed with COPD for the first time based on spirometry outcomes)

A similar picture was obtained for the prevalence of COPD among respondents ≥40 years old (Fig. 2). The estimated prevalence of COPD diagnosed by spirometry was: 47.3 (95% CI 29.9–70.8) per 1000 in Ukraine, 114.1 (95% CI 87.3–145.6) per 1000 in Kazakhstan, and 60.1 (95% CI 40.3–85.7) per 1000 in Azerbaijan; compared to the prevalence of previously diagnosed COPD which was 14.7 (95% CI 5.9–30.1) per 1000 in Ukraine, 26.5 (95% CI 14.2–44.9) per 1000 in Kazakhstan, and 8.6 (95% CI 2.3–21.9) per 1000 in Azerbaijan.

**Fig. 3:**
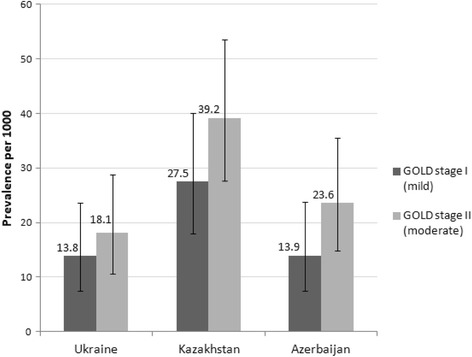
Point prevalence of COPD (diagnosed by spirometry) by GOLD stage. The prevalence of various GOLD stages of COPD was calculated per 1000 persons and expressed with 95% confidence intervals, for *COPD diagnosed by spirometry* (confirmed by spirometry results based on GOLD Guidelines (2011), i.e. FEV1/FVC <  0.70). There were no respondents diagnosed with COPD III or IV GOLD stages in this study

GOLD stage classification (2011) [5] was used for COPD severity (airflow limitation) estimation. COPD patients identified in this study were distributed between stages I (FEV_1_/FVC <  0.70, FEV_1_ ≥ 80% normal) and II (FEV_1_/FVC <  0.70, FEV_1_ 50–79% normal). In all three participating countries, more patients were found in stage II (18.1 (95% CI 10.6–28.8) per 1000 in Ukraine; 48.7 (95% CI 35.9–64.4) per 1000 in Kazakhstan and 21.4 (95% CI 13.1–32.9) per 1000 in Azerbaijan) (Fig. 3).

**Fig. 4:**
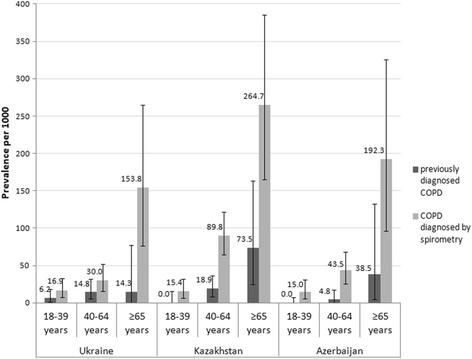
Point prevalence of COPD stratified by age. The prevalence of COPD was calculated per 1000 persons and expressed with 95% confidence intervals in three age groups: 18–39, 40–64, and ≥65 years old, for *previously diagnosed COPD* (when self-reported by the respondent while completing the study questionnaire) and *COPD diagnosed by spirometry* (confirmed by spirometry results based on GOLD Guidelines (2011), i.e. FEV_1_/FVC <  0.70)

A higher prevalence of previously diagnosed COPD was observed in the age group ≥65 years old compared to the younger age groups in Kazakhstan and Azerbaijan: 73.5 (95% CI 24.3–163.3) and 38.5 (95% CI 4.7–132.1) per 1000 respectively. However, in Ukraine, the highest prevalence was observed in the 40–64 years old age group: 14.8 (95% CI 5.4–31.9) per 1000 (Fig. 4). A higher prevalence of COPD diagnosed by spirometry was observed in the population aged ≥65 years old compared to younger age groups in Ukraine, Kazakhstan and Azerbaijan: 153.8 (95% CI 76.3–264.8), 264.7 (95% CI 165.0–385.7) and 192.3 (95% CI 96.3–325.4) per 1000 respectively.

**Fig. 5:**
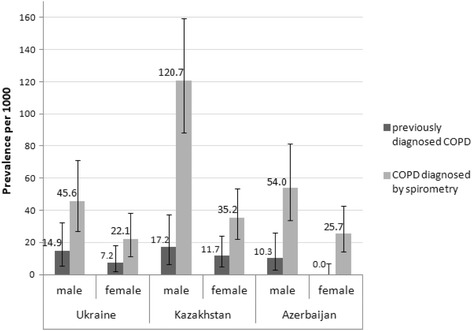
Point prevalence of COPD stratified by gender. The prevalence of COPD was calculated per 1000 persons and expressed with 95% confidence intervals, among men and women, for *previously diagnosed COPD* (when self-reported by the respondent while completing the study questionnaire) and *COPD diagnosed by spirometry* (confirmed by spirometry results based on GOLD Guidelines (2011), i.e. FEV_1_/FVC <  0.70)

**Fig. 6:**
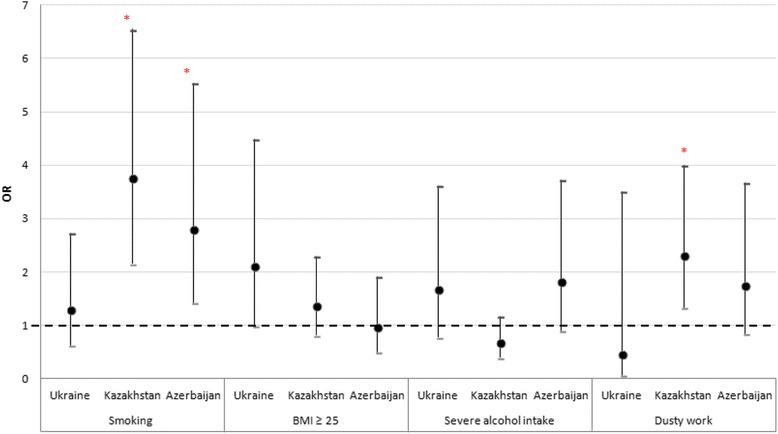
Association between risk factors and COPD diagnosed by spirometry. Odds ratios [OR] and 95% confidence intervals for OR are presented for each potential risk factor. Asterisk (*) denotes as statistically significant association between risk factor and COPD diagnosed by spirometry (*p* < 0.05). For tuberculosis in anamnesis, a significant association was found in Ukraine, but not included in the figure due to the high OR value: 32.393 (CI 4.403–238.330)

In all three participating countries, COPD was more common in men. This applied to both previously diagnosed COPD and COPD confirmed based on spirometry. The prevalence of previously diagnosed COPD was 14.9 (95% CI 5.5–32.2) per 1000 in men vs 7.2 (95% CI 2.0–18.3) per 1000 in women in Ukraine; 17.2 (95% CI 6.4–37.2) per 1000 in men vs 11.7 (95% CI 4.7–24.0) per 1000 in women in Kazakhstan and 10.3 (95% CI 2.8–26.1) per 1000 in men vs 0.0 (95% CI 0.0–6.8) in women in Azerbaijan. The prevalence of COPD diagnosed by spirometry was 45.6 (95% CI 27.2–71.1) per 1000 in men vs 22.1 (95% CI 11.4–38.2) per 1000 in women in Ukraine; 120.7 (95% CI 88.4–159.6) per 1000 in men vs 35.2 (95% CI 21.9–53.3) per 1000 in women in Kazakhstan and 54.0 (95% CI 33.7–81.3) per 1000 in men vs 25.7 (95% CI 14.1–42.8) per 1000 in women in Azerbaijan (Figs. 5 and 6).

The ethnicity-specific prevalence of COPD was estimated only in Kazakhstan, because most Ukrainians and Azerbaijanians were Caucasians. Generally, in Kazakhstan, the prevalence of COPD in Caucasians was slightly higher than in Asians, both for previously diagnosed COPD (17.2 (95% CI 6.3–37.1) per 1000 in Caucasians vs 11.8 (95% CI 4.8–24.2) per 1000 in Asians), and for COPD diagnosed by spirometry (68.8 (95% CI 44.6–100.6) per 1000 in Caucasians vs 64.1 (95% CI 45.7–86.9) per 1000 in Asians).

### Risk factors associated with COPD

The relationship between the presence of COPD and smoking status (current/past smoker), alcohol intake (severe alcohol intake), BMI (BMI ≥ 25 kg/m^2^), tuberculosis (ever diagnosed), dusty work, and open fire cooking was investigated and statistical significance was found between COPD and smoking in Kazakhstan (OR 3.756 (CI 2.156–6.543) *p *< 0.001) and Azerbaijan (OR 2.808 (CI 1.423–5.542) *p* = 0.002). Tuberculosis was a significant risk factor associated with COPD in Ukraine (OR 32.393 (CI 4.403–238.330) *p* < 0.001). Dusty work might increase the appearance of COPD in Kazakhstan (OR 2.306 (CI 1.328–4.002) *p* = 0.002)

### COPD assessment test and the modified Medical Research Council dyspnoea scale

The CAT™ and mMRC dyspnoea scale were assessed in respondents with COPD diagnosed by spirometry (COPD population) and those without COPD (non-COPD population). As seen in Table 5, the total CAT™ score and intensity of dyspnoea/shortness of breath by mMRC were significantly higher among respondents with COPD than in the non-COPD population in all countries. In all non-COPD respondents and COPD respondents in Kazakhstan, the median CAT™ score did not exceed 5 points, which corresponds to the upper limit of normal in healthy non-smokers, and in the COPD population of Ukraine and Azerbaijan the median CAT™ score corresponds to a low (< 10) impact of COPD on health status [12]. According to the mMRC dyspnoea scale, 70–80% respondents without COPD and only half of COPD respondents had no dyspnoea (grade 0).

**Table 5:** CAT™ and mMRC Dyspnoea Scale in COPD and non-COPD population

	Ukraine		Kazakhstan		Azerbaijan	
	Non-COPD	COPD	Non-COPD	COPD	Non-COPD	COPD
CAT™ total score						
median [25–75-percentiles]	2 [0–6]	6 [2–13]	2 [0–5]	4 [2–9]	3 [0–8]	8 [2–14]
*p*-value^a^	0.003		< 0.001		0.004	
mMRC grade, %						
Grade 0	79.4%	50.0%	70.0%	49.2%	75.2%	45.7%
Grade 1	16.1%	43.3%	25.5%	36.5%	22.2%	34.3%
Grade 2	2.4%	6.7%	3.7%	6.3%	1.7%	11.4%
Grade 3	0.9%	0.0%	0.6%	6.3%	0.4%	8.6%
Grade 4	0.4%	0.0%	0.0%	0.0%	0.0%	0.0%
Missing^b^	0.8%	0.0%	0.2%	1.6%	0.0%	0.0%
*p*-value^a^	< 0.001		< 0.001		< 0.001	

^a^two-sided Mann-Whitney U test for a comparison between COPD and non-COPD populations

^b^Respondents with ‘missing’ are not included in calculating *p*-values

COPD population: respondents with COPD diagnosed by spirometry (post-dose FEV_1_/FVC < 0.70)

Non-COPD population: respondents without COPD diagnosed by spirometry (post-dose FEV_1_/FVC ≥ 0.70)

### Co-morbidities

While completing the study questionnaire the respondents were asked to report the presence of other chronic medical conditions except for respiratory diseases. Co-morbidities were reported by 44.2% of respondents in Ukraine, 23.5% respondents in Kazakhstan and 54.6% respondents in Azerbaijan. The respondents with COPD diagnosed by spirometry (COPD population) were compared to the rest of the respondents (non-COPD population) by the rate of co-morbidities. In all participating countries, the number of subjects that reported suffering from a chronic health condition was higher in the COPD population compared to the non-COPD population: 57% vs 43% in Ukraine, 51% vs 27% in Kazakhstan, and 74% vs 54% in Azerbaijan, respectively (Table 6).

**Table 6:** Co-morbidities in COPD and non-COPD population

	Ukraine			Kazakhstan			Azerbaijan		
	Non-COPD*N* = 909	COPDN = 30	*p*-value^a^	Non-COPD*N* = 882	COPDN = 63	*p*-value^a^	Non-COPD*N* = 898	COPDN = 35	*p*-value^a^
Any co-morbidity	42.5%	56.7%	0.136	26.9%	50.8%	< 0.001	53.8%	74.3%	0.023
Hypertension	14.0%	40.0%	0.001	25.5%	36.5%	0.074	16.8%	34.3%	0.012
Diabetes	3.4%	6.7%	0.621	3.4%	7.9%	0.078	5.2%	8.6%	0.428
Cardiovascular disease	6.0%	26.7%	˂0.001	7.8%	22.2%	0.001	3.5%	22.9%	˂0.001
Abnormal blood lipids	4.9%	13.3%	0.062	11.7%	15.9%	0.421	1.3%	0.0%	1.000
Depression	0.7%	0.0%	1.000	0.6%	0.0%	1.000	1.9%	2.9%	1.000
Anxiety	0.2%	0.0%	1.000	0.6%	0.0%	1.000	2.6%	5.7%	0.240
Osteoporosis	0.7%	0.0%	1.000	1.6%	3.2%	0.618	0.6%	0.0%	1.000
Tuberculosis	0.2%	6.7%	0.006	2.3%	0.0%	0.391	1.4%	2.9%	1.000
Pneumonia	19.4%	36.7%	0.024	14.9%	31.7%	0.001	5.9%	17.1%	0.019

^a^two-sided Pearson Chi-Square test for a comparison between COPD and non-COPD populations

COPD population: respondents with COPD diagnosed by spirometry (post-dose FEV_1_/FVC < 0.70)

Non-COPD population: respondents without COPD diagnosed by spirometry (post-dose FEV_1_/FVC ≥ 0.70)

A history of pneumonia was significantly more frequent in the COPD population than in the non-COPD population in all investigated countries (36.7% vs 19.4%, *p* = 0.024 in Ukraine, 31.7% vs 14.9%, *p* = 0.001 in Kazakhstan and 17.1% vs 5.9%, *p* = 0.019 in Azerbaijan). The same results were obtained for a previous cardiovascular disease (26.7% vs 6.0%, *p *< 0.001 in Ukraine, 22.2% vs 7.8%, *p* = 0.001 in Kazakhstan and 22.9% vs 3.5%, *p*˂0.001 in Azerbaijan in COPD and non-COPD populations respectively). Additionally, hypertension occurred significantly more often in the COPD population compared to the non-COPD population in Ukraine (40.0% vs 14.0%, *p* = 0.001) and Azerbaijan (34.3% vs 16.8%, *p* = 0.012). In Kazakhstan, hypertension was also more frequently observed in the COPD population (36.5%) compared to the non-COPD population (25.5%) but the difference was not shown to be significant (*p* = 0.074) (Table 6).

### Adverse events

In all three participating countries, no serious adverse events (related to participation in the study) were recorded during the study period. Non-serious adverse events were not collected, as there was no investigational drug administration in this study.

## Discussion

The CORE study is, to our knowledge, the first epidemiological study conducted to evaluate the prevalence and burden of COPD in CIS countries using a standardized methodology. The study showed that COPD prevalence reported by respondents ‘previously diagnosed’ with COPD was 10.4, 13.8 and 4.3 per 1000 in Ukraine, Kazakhstan, and Azerbaijan, respectively, but the prevalence of COPD diagnosed by spirometry was much higher, 31.9, 66.7 and 37.5 per 1000 respectively. This can be explained by under diagnosis of the disease in CIS countries. Therefore, the majority of respondents with COPD were diagnosed for the first time in this study. As highlighted by Pasko (2002), COPD is often under-reported which could explain why official data on COPD prevalence are up to 10-fold lower than actual population data [14]. The prevalence of COPD observed in Kazakhstan was high compared to Ukraine and Azerbaijan; one possible explanation for this may be the relatively poor ecological conditions in Almaty. This city is surrounded by high mountains (3000–5000 m) and experiences little wind, whereas the climate in Kiev and Baku is windy and there is a river or sea. However, this assumption needs further investigation.

A statistically significant relationship was shown between smoking and COPD in Kazakhstan and Azerbaijan; tuberculosis and COPD in Ukraine; and dusty work and COPD in Kazakhstan. Co-morbidities were significantly more frequent in the COPD population compared to the non-COPD population.

In all countries studied, the frequency of COPD diagnosed by spirometry had a tendency to increase with age (peak prevalence occurred at the age of 65 years and older). This trend has been observed in many studies [15–17] and can be explained by greater exposure to risk factors and physiological decrease in lung function with age. In epidemiological studies, high prevalence of COPD in the elderly has been reported widely, in age groups > 50 years [18], 55–70 years [19], ≥ 60 years [20], 75–84 years [21], ≥ 70 years [22].

In all three countries, COPD was more prevalent in men (both diagnosed by spirometry and previously diagnosed), in line with numerous other studies [7, 15]. This fact can probably be explained by different susceptibility associated with gender or by increased exposure to risk factors in men (smoking, occupational hazards, etc.). A low rate of previously reported COPD compared to spirometry findings was also noted in other studies [15, 23]. This could indicate a significant underestimation of COPD prevalence in these countries, probably related to late visits to doctors (because of limited accessibility to primary medical care or non-specific and/or mild symptoms at early stages of disease) and lack of population screening. It should be noted that not all participants with previously diagnosed COPD underwent spirometry (especially in Kazakhstan and Azerbaijan), so the real prevalence of spirometry changes relevant to COPD may be substantially higher.

Ethnicity-related differences were assessed in Kazakhstan only and revealed a higher prevalence of COPD (both diagnosed by spirometry and previously diagnosed by a physician) in Caucasians compared to Asians, together with more prominent symptoms and spirometry changes. Other studies have mentioned that genetic factors are probably involved in the decreased COPD risk observed in Asian [24].

### Comparison with published literature

The prevalence of previously diagnosed COPD shown in the present study is low compared to estimates provided by previous studies (42 in Brazil [25], 57 in Greece [15], 88 in The Netherlands [23] and 222 in Russia [26] per 1000). Sweden was the only country where the reported prevalence was comparable to the present study (18 per 1000) [21]. This can possibly be explained by peculiarities of the healthcare systems. In the CIS countries, a big issue is the lack of COPD knowledge and the attitude to this disease within the population (people do not take a “simple” cough or dyspnea seriously and therefore do not visit a doctor). Another major problem may be related to low access to primary care services; in all the countries that took part in this study primary care is underfinanced and underdeveloped. Under-reporting of COPD may reflect the lack of COPD knowledge not only among patients, but also among healthcare workers, especially general practitioners and internists, who are the primary contact between the healthcare system and the population. For example, in Kazakhstan as early as 10–15 years ago, COPD was considered an “exotic” disease; a COPD diagnosis would be given in specialized institutions, not by primary care physicians. Nowadays COPD is much more well known. However, some public health problems may still exist; for example, patients receive medications for COPD treatment free of cost which may force health authorities to regulate the number of COPD patients registered in primary and specialty care. The forth reason for COPD under-reporting may be related to low availability of spirometry and its low quality in primary care hospitals. From a public health perspective, another important issue is that all respiratory diseases are collected in one statistical pool; there is no separate registration of COPD, asthma, allergic rhinitis and other non-infectious respiratory diseases in the CIS countries.

The COPD prevalence estimates based on spirometry reported in this study were similar to the data published in previous studies (45 in Spain [16], 42 in USA [19] and 37 in United Arab Emirates [22] per 1000). Some other studies reported higher estimates (240 (The Netherlands) [23], 218 (Russia) [26], 197 (Uruguay) [20], 184 (Greece) [15], 162 (Uganda) [27], 162 (Sweden) [21], 142 (Portugal) [28], 134 (Korea) [17], 78 (Mexico) [20] and 71 (Vietnam) [18] per 1000). The variability in prevalence estimates could reflect true differences or be due to the different methodologies used in different studies. Study design aspects such as differences in the rigor of case ascertainment (for example, different diagnostic criteria) or the over-representation of a high-risk sub-population in the study (such as another age range of participants or percentage of smokers) or even differences in healthcare systems (the availability of medical care) could have led to variations in estimates. However, different nations or regions may have a truly increased or decreased burden of the disease as a result of true biological phenomena. It should be noted that in the Russian study (GARD) spirometry was performed only for participants with suspected COPD, so the prevalence may be overstated; in addition, a significantly higher prevalence of previously diagnosed COPD in the Russian study is probably due to different diagnostic criteria. One study estimated the prevalence of COPD firstly diagnosed by spirometry [15]. In line with our study, most cases of COPD diagnosed by spirometry were firstly diagnosed, and prevalence of firstly diagnosed COPD was higher than for the previously diagnosed COPD confirming the observation that COPD is often under-reported.

### COPD characteristics

The majority of respondents with COPD diagnosed by spirometry in this study had mild/moderate COPD; there were no respondents with GOLD stages III or IV. The median CAT™ score did not exceed 10 points, which corresponds to a low impact of COPD on health status. Almost half of respondents with COPD did not have dyspnoea/shortness of breath by mMRC scale. At the same time, clear statistically significant differences for CAT™ and mMRC scale were obtained between COPD and non-COPD respondents that additionally confirm the validity of these instruments for evaluation of COPD in research studies and routine use.

### Co-morbidities

As for co-morbid conditions, in Ukraine the participants more often reported unburdened anamnesis, than in Kazakhstan and especially in Azerbaijan. A high rate of arterial hypertension was observed, followed by other cardiovascular diseases, diabetes mellitus and blood lipid abnormalities. In other studies, cardiovascular diseases were also the most common co-morbidities [29] and were recorded more frequently than in this study.

As expected, the most significant correlation was found between COPD and cardiovascular diseases. The rate of hypertension was also higher among respondents with COPD compared to the non-COPD population (although this difference was only statistically significant in Ukraine and Azerbaijan). It is well established, that COPD is a precursor to cardiovascular disease development and/or its aggravation [30, 31]. The association between a history of pneumonia and COPD confirms available data. Pneumonia and COPD can aggravate each other. One study indicated that previous exacerbations of pneumonia are significantly associated with a higher rate of COPD exacerbation [32]. Another study confirmed that COPD increases mortality in patients with pneumonia [33].

### Strengths and limitations

This study has several strengths. It is a multi-national, cross-sectional, population-based study with a large sample size using consistent methodology across all countries, providing a standardized measure of prevalence in the CIS countries. In addition, the case definition of COPD used is based on both (i) self-reported diagnosis and (ii) diagnosis confirmed by spirometry. Furthermore, severity of COPD has been assessed based on the validated international GOLD guidelines. As it is the first time such an epidemiological study has been conducted in CIS countries, this study could facilitate increased recognition of COPD in CIS countries and allow preparation of educational interventions to optimize management of patients with COPD. Whereas many previously published studies on COPD concern patients only over 40 years of age, this study evaluates COPD prevalence in the overall adult population.

We acknowledge that the current study has several limitations. The method of district and street sampling may not ensure completely random selection of streets and participants. The relatively small number of COPD patients limits the analysis of risk factors associated with COPD and can limit the power for specific types of within-city analysis, such as detailed subgroup analyses. The subjectivity of the diagnostic criteria based on symptoms can lead to over or under diagnosis. Spirometry was the only objective diagnostic measure in the present study and its results were reviewed centrally, but difficulties encountered when conducting spirometry can affect the results of this procedure. Additionally, the physiological decrease in lung function in the elderly may influence the estimation of COPD prevalence stratified by age, taking into account that fixed ratio FEV_1_/FVC was used as the diagnostic criteria for COPD in this study.

The city population may not be representative of each country in general, because risk factors and healthcare provision (including the availability of medical care) may vary widely across the country. In particular, the results may only reflect the situation in urban areas and not represent the whole country, since rural areas could have different levels of healthcare provision and accessibility to medical care and different living and working conditions for the people who live in these areas. Finally, some data are missing due to a lack of relevant information from participants, as a lot of study data were collected from respondents’ interview.

## Conclusion

In conclusion, in Ukraine, Kazakhstan and Azerbaijan the prevalence of COPD diagnosed by spirometry is significantly higher than the prevalence self-reported by respondents and/or based on anamnesis, demonstrating that COPD is under-reported in these countries. Compared to other countries, the COPD prevalence estimates in these CIS countries were relatively low. Factors such as limited funding from the government, lack of COPD knowledge, attitude within the population and of primary care physicians, and low access to high-quality spirometry may play a role in the under-reporting of COPD in these countries. A higher rate of COPD prevalence was observed in Kazakhstan (Almaty) compared to Ukraine (Kiev) and Azerbaijan (Baku) likely due to poor ecological conditions, but this assumption needs further investigation. The information provided in this paper will be helpful for healthcare policy makers in CIS countries to instruct COPD disease management and prevention strategies and allocate healthcare resources accordingly.

## Abbreviations

ATS: American Thoracic Society
BMI: Body mass index
CAT™: COPD Assessment Test
CI: Confidence interval
CIS: Commonwealth of Independent States
COPD: Chronic Obstructive Pulmonary Disease
ERS: European Respiratory Society
FEV_1_: Forced Expiratory Volume in one second
FVC: Forced Vital Capacity
GARD: Global Alliance against Chronic Respiratory Diseases
GOLD: Global Initiative for Chronic Obstructive Lung Disease
GSK: GlaxoSmithKline
mMRC: Modified Medical Research Council
OR: Odds Ratio
PaCO_2_: Arterial partial pressure of carbon dioxide
PaO_2_: Arterial partial pressure of oxygen
SD: Standard Deviation
